# Management of Neonatal Diabetes due to a *KCNJ11* Mutation with Automated Insulin Delivery System and Remote Patient Monitoring

**DOI:** 10.1155/2023/8825724

**Published:** 2023-08-23

**Authors:** Ming Yeh Lee, Anna L. Gloyn, David M. Maahs, Priya Prahalad

**Affiliations:** ^1^Division of Pediatric Endocrinology, Stanford University School of Medicine, Stanford, CA, USA; ^2^Stanford Diabetes Research Center, Stanford University School of Medicine, Stanford, CA, USA

## Abstract

Neonatal diabetes mellitus (NDM) is a monogenic form of diabetes. Management of hyperglycemia in neonates with subcutaneous insulin is challenging because of frequent feeding, variable quantity of milk intake with each feed, low insulin dose requirements, and high risk for hypoglycemia and associated complications in this population. We present a case of NDM in a proband initially presenting with focal seizures and diabetic ketoacidosis due to a pathologic mutation in the beta cell potassium ATP channel gene *KCNJ11* c.679G > A (p.E227K). We describe the use of continuous glucose monitoring (CGM), insulin pump, automated insulin delivery system, and remote patient monitoring technologies to facilitate rapid and safe outpatient cross-titration from insulin to oral sulfonylurea. Our case highlights the safety and efficacy of these technologies for infants with diabetes, including improvements in glycemia, quality of life, and cost-effectiveness by shortening hospital stay.

## 1. Introduction

Neonatal diabetes mellitus (NDM) occurs in approximately 1 in 90,000 to 160,000 live births [1]. Genetic testing provides the diagnosis and guides clinical therapy. Mutations affecting the pancreatic beta cell potassium ATP channel genes (*KCNJ11* and *ABCC8*) are common in neonatal diabetes, and these patients can often be transitioned from insulin to oral sulfonylurea therapy [2]. In a healthy pancreatic beta cell, glucose enters via a glucose transporter, where it is metabolized, resulting in a change in the ratio between ADP and ATP which results in closure of ATP-dependent potassium (K_ATP_) channels leading to membrane depolarization and activation of voltage-dependent calcium channels. The subsequent influx of calcium is the trigger for insulin release. Heterozygous activating mutations in the genes encoding for the two subunits of the K_ATP_ channel (*KCNJ11* or *ABCC8*) result in an inability of ATP to lead to channel closure which prevents insulin secretion [2]. Sulfonylureas close the K_ATP_ channel by an ATP-independent mechanism, allowing for insulin to be released from the beta cell [1]. Patients with this genetic etiology for their diabetes can be treated with oral sulfonylureas rather than insulin with improvements in both glycemia and quality of life (QoL) [3–5].

Management of NDM with subcutaneous insulin is challenging because of frequent feeding, variable quantity of milk intake with each feed, low insulin dose requirements, and high risk for hypoglycemia and associated complications in infants. Various technologies including continuous glucose monitors (CGMs), insulin pumps, automated insulin delivery (AID) systems, and remote patient monitoring (RPM) have improved glycemia and QoL for youth with type 1 diabetes [6]. These technologies are likely to improve NDM management although they have not been well studied nor have FDA approval for use in this population. CGMs have been utilized for glucose monitoring in neonates with conditions such as NDM and congenital hyperinsulinism [7]. CGMs are beneficial for alerting impending hypoglycemia, allowing caregivers to recognize and treat hypoglycemia in neonates, who otherwise have difficult to recognize hypoglycemia symptoms. Insulin pumps may benefit NDM management by (1) allowing precise delivery of small insulin doses compared to subcutaneous injections, (2) allowing flexibility of frequent insulin boluses for hyperglycemia correction or carbohydrate coverage without additional injections, and (3) minimizing the potential for stacking of insulin boluses, leading to hypoglycemia with the insulin-on-board feature [1].

AID systems combine a CGM, an insulin pump, and a dosing algorithm for insulin delivery. Guidelines from the International Society for Pediatric and Adolescent Diabetes and American Diabetes Association strongly recommend the use of AID systems for youth with diabetes, as these systems improve CGM time in range (TIR, glucose 70–180 mg/dL) [8, 9]. Previously, AID systems have been used for the management of hyperglycemia in extremely preterm infants in the neonatal intensive care unit, demonstrating improved TIR and optimized nutritional intake without increasing the risk of hypoglycemia [10]. However, outpatient use of AID systems for management of NDM has not been reported. RPM allows flexible and timely interventions for youth with diabetes without requiring additional clinic visits [11]. Historically, titration from insulin to sulfonylurea therapy has occurred rapidly in an inpatient setting or more slowly in an outpatient setting. Our case demonstrates that early diagnosis of sulfonylurea-responsive NDM and incorporation of AID and close guidance of experienced pediatric endocrinologists led to safe, fast, and effective transition to sulfonylurea therapy and glycemic control in the outpatient setting, while improving patient and family's QoL.

## 2. Case Presentation

A 2-month-old female presented to the emergency department with left-sided focal seizures. She had no infectious symptoms and had been feeding and acting normally. She was born full term with history of small for gestational age (birth weight of 2205 g, 0.6 percentile). At 6 weeks of age, she had good catch up growth to 3459 g (1.58 percentile) and normal development. Her physical exam was normal with no dysmorphic features and no focal neurological deficits. Initial laboratory evaluation was notable for glucose of 604 mg/dL and mild diabetic ketoacidosis (DKA) with venous pH 7.28, beta-hydroxybutyrate 4.2 mmol/L, elevated hemoglobin A1c 8.5%, and low c-peptide 0.45 ng/ml. Head CT showed no acute intracranial abnormalities. Brain MRI was notable for foci of restricted diffusion in the bilateral frontal cerebral white matter, bilateral cerebellar white matter, and right putamen with surrounding signal abnormality. She was admitted to the pediatric intensive care unit for intravenous insulin and fluids based on the institutional DKA protocol and for seizure monitoring and treatment. She remained on broad-spectrum antibiotics until infectious workup was negative. Continuous electroencephalogram (EEG) demonstrated focal right temporal seizures that were associated with left facial and eyelid twitching. EEG also demonstrated focal slowing in the right hemisphere, which is a nonspecific indicator of cerebral dysfunction from a wide variety of potential etiologies. Pediatric neurology and radiology considered these findings most likely related to severe infantile hyperglycemic injury.

## 3. Treatment, Outcome, and Follow-Up

**Figure 1:**
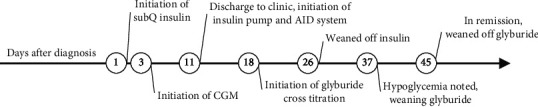
Timeline of clinical events.

On day 2 of admission, DKA resolved and she transitioned from intravenous to subcutaneous insulin (Figure 1). Genetic panel for monogenic diabetes was sent. Since her clinical presentation was highly suspicious for NDM and genetic testing was still pending, we initiated discharge planning for potential long-term insulin requirement. She was initiated on the Dexcom G6 (Dexcom, San Diego, CA) CGM for glucose monitoring and family received teaching for diabetes management. While insulin pump and AID system were recognized as tools for aiding glycemia, barriers to inpatient initiation of technologies included lack of hospital policy and lack of familiarity and comfort from primary providers and nursing staff. Thus, initiation of insulin pump and AID system was deferred until an outpatient clinic visit.

**Figure 2:**
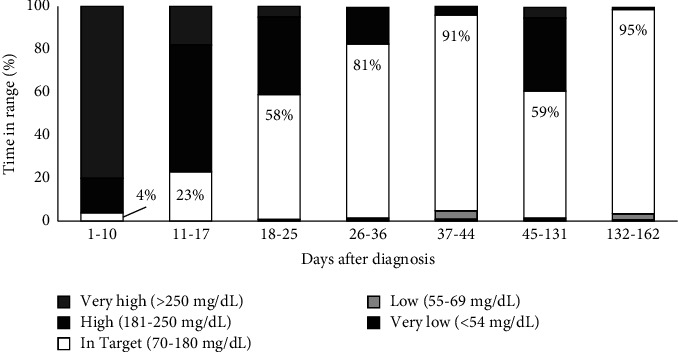
CGM glucose pattern summary data during various periods of treatment.

Subcutaneous insulin doses were initiated during admission using basal-bolus strategy with U100 Lantus once daily and diluted U10 Humalog for hyperglycemia correction every 4 hours. Doses were titrated daily during the first week of diagnosis, to total daily insulin dose of 5 units (1.4 units/kg/day). Blood glucose target was set at 200 mg/dL due to the risk for complications from hypoglycemia. Glycemia was poor during the initial week on subcutaneous insulin regimen, with mean glucose 300 mg/dL, standard deviation 59 mg/dL, CGM TIR (glucose 70-180 mg/dl) 4%, time in hyperglycemia (glucose above 180 mg/dL) 96%, and no hypoglycemia (glucose less than 70 mg/dL) (Figure 2). Challenges to management include limitation of subcutaneous insulin dose increments, insulin dose injection volume, risk of hypoglycemia, frequency of feeding, and variable amount of feeding due to changing mental status while titrating antiepileptics. Seizure control required 4 antiepileptics.

By day 9 after diagnosis, patient returned to baseline mental status, was feeding well, and was seizure free on a stable regimen of antiepileptics. She was discharged directly to the diabetes clinic for initiation of the t:slim X2 insulin pump (Tandem, San Diego, CA) with Control-IQ technology for AID. The pump was loaded with dilute U10 Humalog, with basal rate set to 0.1 unit/hour, carbohydrate ratio of 1 unit per 60 g carbohydrates, and correction factor of 1 unit to glucose of 400 mg/dL. The pump was set to “exercise mode” of control-IQ to enable higher glucose target. CGM and insulin data are shared with the diabetes team via Dexcom Clarity (Dexcom, San Diego, CA) and t:connect (Tandem, San Diego, CA) web portals, respectively, for RPM in between clinic visits. Glycemia was improved on AID compared with multiple daily injection insulin. Her mean glucose was 211 ± 43 mg/dL, TIR 23%, hyperglycemia 77%, and no hypoglycemia (Figure 2).

Week 2 after presentation, genetic testing identified a *KCNJ11* c.679G > A (p.E227K) heterozygous mutation, a pathologic variant in the gene encoding for potassium ATP channel in pancreatic beta cells [1]. Reflex genetic testing on the patient's parents were negative for the same pathologic variant, indicating that this was a *de novo* mutation. Rare pathogenic mutations in *KCNJ11* are a cause of multiple disorders of insulin secretion with the direction of effect depending on whether mutations result in a loss or gain of function. Heterozygous activating mutations can cause several varieties of NDM, including both permanent and transient forms, with the functional severity of the mutation influencing the clinical presentation [2]. Patients with *KCNJ11* activating mutations are typically responsive to treatment with oral sulfonylureas with improvements in both glycemia and QoL due to the ease of administrating oral medication versus insulin injections multiple times daily [1, 3–5]. Several studies indicate that commencing treatment at the earliest opportunity may also improve neurodevelopmental outcomes in sulfonylurea-responsive patients [1].

**Figure 3:**
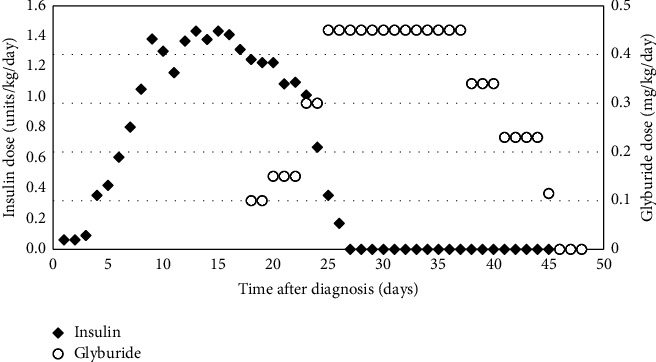
Timeline of insulin to glyburide cross-titration. Glyburide dose was quickly up-titrated as an outpatient every 2-3 days to maximal effect over 1 week. Insulin was safely weaned off 9 days after initiation of glyburide.

**Figure 4:**
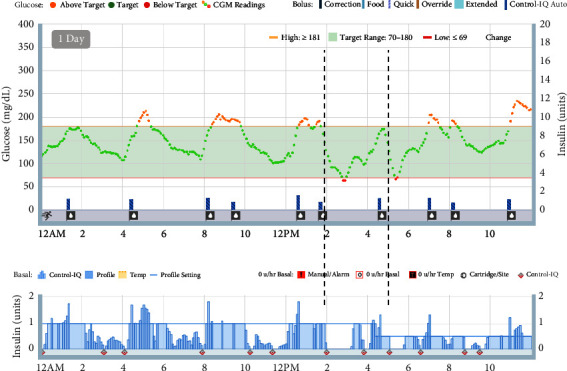
Representative CGM and insulin pump data for 24 hours during glyburide cross-titration. Two dashed lines mark anticipated hypoglycemia and appropriate suspension of insulin delivery from the pump.

Day 18 after diagnosis, we initiated cross-titration from insulin to glyburide, an oral sulfonylurea, based on a published protocol [1]. Typically, outpatient cross-titration of oral sulfonylurea is titrated weekly due to the risk of hypoglycemia when insulin requirement decreases. However, we were able to safely and rapidly achieve outpatient cross-titration using the AID system coupled with RPM (Figure 3). The AID system minimizes risk of hypoglycemia by suspending insulin delivery in response to hypoglycemia or anticipated hypoglycemia. Asynchronous RPM with pediatric endocrinologist reviewing CGM and AIDs data every 2-3 days facilitated frequent outpatient dose adjustments. By day 9 of cross-titration, glyburide was titrated to 0.45 mg/kg/day and insulin was weaned off. This was a similar timeline as inpatient cross-titration, and much more rapid compared to outpatient cross-titration [1]. During cross-titration, glycemia continued to improve (mean glucose 169 ± 47 mg/dL, TIR 58%, hyperglycemia 41%, and hypoglycemia <1%). Review of AID data showed that insulin delivery suspended appropriately in anticipation of hypoglycemia (Figure 4). On glyburide monotherapy, glycemia was achieved (mean glucose 130 ± 38 mg/dL, TIR 86%, hyperglycemia 10%, and hypoglycemia <4%). Week 5 after diagnosis, she had intermittent preprandial hypoglycemia suggestive of clinical remission from diabetes. Glyburide was weaned off with subsequent normoglycemia (mean glucose 110 ± 26 mg/dL, CGM 95%, hyperglycemia 1%, and hypoglycemia <4%) (Figure 2). Overall, this clinical course is consistent with an activating mutation in *KCNJ11* leading to transient NDM (TNDM). A large proportion of TNDM cases subsequently relapse and will require insulin therapy, most often during adolescence [1]. Thus, it remains important to monitor for signs and symptoms of hyperglycemia and periodically monitor hemoglobin A1c even if she is off insulin and sulfonylurea.

## 4. Discussion

Some activating mutations in *KCNJ11* have been associated with developmental delay, epilepsy, and neonatal diabetes (DEND) and intermediate DEND (iDEND) syndrome. In addition to the pancreatic islet, *KCNJ11* is also expressed in neurons and skeletal muscles; thus, altered activity of K_ATP_ channels in these tissues may explain the neurodevelopmental features [2]. The *KCNJ11* p.E227K mutation has been reported in patients with a range of diabetes presentation including neonatal and later-onset phenotypes [12–14]. It has been previously described in a patient with TNDM, axial hypotonia during infancy, and moderate neurodevelopmental delay in language and mathematical reasoning during childhood [15]. However, this mutation has not been associated with seizures. In children with type 1 diabetes, long-term changes in brain structure and cognitive function have been associated with hyperglycemia [16]. Thus, our patient's initial seizures may have been provoked by hyperglycemic brain injury. She has been followed by pediatric neurology and developmental-behavioral pediatrics with no concerns about development, no more clinical seizures, and a normal EEG while weaning antiepileptics. She will continue to follow with neurology for neurodevelopmental monitoring. Given her history of seizures, it is important to closely manage her blood glucoses to avoid further neurologic insult from hyperglycemia or hypoglycemia.

In summary, our experience demonstrates that management of infants with NDM using the AID system and RPM under close guidance of experienced pediatric endocrinologists can lead to safe and effective glycemic control and at home transition to sulfonylurea therapy. Benefits of these technologies include improved TIR by minimizing hyperglycemia and hypoglycemia and improved QoL for the family. The strategy of using AID systems coupled with RPM further allows frequent outpatient medication titration, thus reducing the length of hospital stay and cost. We advocate that all children should be allowed to use these technologies inpatient and outpatient when proper supervision by experienced medical providers is available. Our report has broad implications as hospital policies and infrastructure need to be reviewed and updated regularly to prevent hindering optimal patient care through application of novel technologies.

## Data Availability

The data used to support the findings of this study are available from the corresponding author upon request.

## Additional Points

(i) Availability of rapid genetic testing allows possibility of early transition from insulin to sulfonylurea therapy for patients with neonatal diabetes caused by *KCNJ11* and *ABCC8* mutations. (ii) Glycemic management of neonates with diabetes is challenging due to limitations in subcutaneous insulin dose increments, insulin dose injection volume, high risk of hypoglycemia, high frequency of feeding, and variable amount of feeding. (iii) Management of neonatal diabetes using AID systems and RPM under close guidance of experienced pediatric endocrinologists can lead to safe and effective glycemia by minimizing both hyperglycemia and hypoglycemia.
